# A multi-toxicity deep learning approach for normal tissue complication probability modelling in head and neck cancer patients receiving radiotherapy

**DOI:** 10.1016/j.radonc.2026.111486

**Published:** 2026-03-17

**Authors:** D.C. MacRae, L. van der Hoek, S.P.M. de Vette, H. Neh, A.C. Moreno, C.D. Fuller, J.A. Langendijk, M.A. Valdenegro-Toro, N.M. Sijtsema, P.M.A. van Ooijen, L.V. van Dijk

**Affiliations:** a Department of Radiation Oncology, University Medical Center Groningen, University of Groningen, Groningen, the Netherlands; b Department of Radiation Oncology, The University of Texas MD Anderson Cancer Center, Houston, USA; c Department of Artificial Intelligence, Bernoulli Institute, University of Groningen, the Netherlands

**Keywords:** Multi-toxicity, Normal tissue complication probability, Deep learning, Radiotherapy, Aspiration, Dysphagia, Sticky saliva, Taste alteration, Xerostomia

## Abstract

**Purpose:**

Toxicities after radiotherapy for head and neck cancer (HNC) often co-occur and share underlying mechanisms, yet most conventional and deep learning (DL) NTCP models predict only a single endpoint. By developing DL NTCP models which can predict multiple toxicities simultaneously, this study aimed to capture inter-toxicity relationships to improve prediction performance.

**Methods and materials:**

A multi-institutional cohort of 1,418 HNC patients was used to develop and validate a multi-toxicity (MT) DL model, incorporating 3D dose distributions, CT scans, organ-at-risk segmentations and patient-related features, that simultaneously predicts five toxicities; aspiration, dysphagia, sticky saliva, taste alteration and xerostomia, all evaluated six months after treatment. Results are compared to conventional NTCP models, as well as a set of single-toxicity (ST) 3D DL models.

**Results:**

The MT model outperformed both the conventional and ST models for dysphagia (AUC = 0.83 versus 0.81 and 0.82) and xerostomia (0.80 versus 0.75 and 0.78) prediction on the independent validation cohort. The latter models achieved better performance for sticky saliva (0.72 and 0.71 versus 0.69) and taste alteration (both 0.67 versus 0.71). The MT model achieved a higher AUC on aspiration than the ST model (0.71), but performed as well as the reference model (both 0.74). Within the external validation cohort, all models performed comparably to each other, with the MT model achieving a slightly higher average AUC (0.64) across all endpoints than the conventional and ST models (both 0.63). Sub-analyses revealed that the benefit of the proposed multi-toxicity modelling varied by endpoint.

**Conclusion:**

MT models offer comparable—and in some cases improved—performance over conventional single-endpoint approaches, indicating their promise for NTCP modelling. However, benefits are not uniform across all endpoints, highlighting the importance of considering toxicity-specific features when designing multi-toxicity models.

## Introduction

Radiotherapy plays a key role in the treatment of head and neck cancer (HNC) patients. However, it often results in collateral damage to the surrounding healthy tissues, leading to a range of toxicities that can significantly impact patients’ quality of life both during and after treatment [1–3]. Common radiation-induced toxicities are dysphagia (difficulty swallowing), taste alteration, and xerostomia (dry mouth), which may co-occur and are interrelated in their development [3,4]. Given their clinical impact, there is a pressing need to accurately assess the risk of multiple, co-occurring late toxicities as a whole to support personalized treatment selection and dose optimization [5,6].

Normal tissue complication probability (NTCP) models aim to predict a patient’s risk of developing a specific toxicity. Conventional NTCP models rely on a small number of predictive features; incorporating discrete organ-at-risk (OAR) dose parameters and, in some models, patient characteristics, to estimate toxicity risk [7]. For example, models have used the mean dose to the oral cavity for both dysphagia and taste alteration prediction [8], yet these toxicities depend on different regions within the same OAR—swallowing muscles versus taste buds. Reducing a complex 3D dose distribution into one-dimensional features (e.g. mean dose) discards this clinically relevant information. To overcome these limitations, deep learning (DL) NTCP models, particularly convolutional neural networks (CNNs), have been developed to learn directly from 3D patient images and dose distributions. Within HNC, such models have already shown promise for predicting xerostomia [9,10], dysphagia [11], tube-feeding dependence [12], taste alteration [13], and osteoradionecrosis [14]. However, despite overlaps in toxicity symptoms and their predictors, both conventional and DL NTCP models remain designed for single endpoints and thus overlook known interactions between toxicities. For instance, xerostomia and sticky saliva both have similar predictors; the (mean) dose to the salivary glands [15], while saliva flow is a key factor in taste perception changes, and can also contribute to dysphagia, which in turn increases the risk of aspiration [4,16,17].

Given these interdependencies and overlapping predictors, we propose a DL multi-toxicity modelling approach that uses pre-treatment information to predict multiple late toxicities simultaneously. We hypothesize that modelling these toxicities together will enable a multi-label DL model to capture interactions between their development, ultimately improving predictive performance for each individual toxicity.

This study aims to improve the prediction of HNC toxicities by developing a multi-toxicity DL model on a large cohort of HNC patients, comparing its performance to conventional NTCP modelling approaches as well as single toxicity DL models. Targeting common and clinical influential toxicities, we predict xerostomia, sticky saliva, taste loss, aspiration, and dysphagia 6 months post-radiation therapy simultaneously.

## Materials and methods

### Patients and treatment characteristics

HNC patients with squamous cell carcinoma who received radiation therapy with curative intent at University Medical Centre Groningen (UMCG, 2007–2021, NCT02435576) and MD Anderson Cancer Centre (MDACC, 2015–2021, PA14–0947 data collection, PA11–0809 analysis), and for which at least one of the five toxicity scores was available at six months post-radiotherapy, were included in the current study. We also evaluated a stricter inclusion criterion requiring at least two available toxicity endpoints per patient, to assess whether explicitly enforcing observed co-occurrence was necessary. Exclusion criteria were previous treatment for HNC, surgery in the head and neck region (except for tonsillectomies or laser treatment of small glottic lesions), induction chemotherapy, distant metastasis, age under 18, any fraction dose exceeding 2.4 Gy, not completing all planned treatment fractions, and missing any of the OARs which are used in the reference NTCP models (described below).

Patients in the UMCG cohort were treated with 66–70 Gy to the primary tumour, and a 54.25 Gy elective dose, over 6–7 weeks (33–35 fractions) with or without concurrent platinum-based chemotherapy or cetuximab. Patients in the MDACC cohort typically received a dose of 66–70 Gy to the primary tumour and 56–57 Gy in elective dose over 30–35 fractions, with or without concurrent platinum-based chemotherapy. In both centres, the salivary glands were spared as much as possible during treatment optimisation. After 2010, swallowing structures were also spared at the UMCG [18].

Planning CTs were obtained for each patient in treatment position, upon which OARs were delineated following the guidelines of Brouwer et al. [19] using manual contouring, as well as automatic delineation using deep learning contouring (Mirada) after 2019, for the UMCG cohort and atlas-based auto-contouring (Elekta ADMIRE) for the MDACC cohort [20]. Where necessary, missing OAR contours (described in Appendix A2) were obtained using a deep learning contour algorithm [21]. OAR mean dose values were derived from the dose distributions and aforementioned OAR contours using MATLAB (R2018b).

The UMCG cohort was split into a model development cohort (80%) and independent validation cohort (20%). This split was stratified on contrast-enhanced CTs scans, the presence of metal artefacts within the CT, tumour location and treatment modality (photon- or proton-treatment). The MDACC cohort was used as an external validation set.

### NTCP endpoints

The NTCP endpoints were the following five toxicities, assessed at six months after radiation therapy: patient-rated moderate-to-severe aspiration, sticky saliva, taste loss, and xerostomia, and physician-rated grade 2–4 dysphagia. For the UMCG cohorts, “moderate-to-severe” was defined as the highest two scores on the 4-point Likert scale EORTC QLQ-H&N35 questionnaire [22], while grade 2–4 dysphagia was evaluated according to the CTCAEv4.0 criteria [23].

For the external validation cohort, toxicity ratings were derived from the MD Anderson Symptom Inventory-Head and Neck Module (MDASI-HN, rated on a scale of 0 to 10) [24] and the Performance Status Scale for Head and Neck Scores (PSS-HN, rated out of 100) [25] symptom rating systems. We converted these ratings to the same scale as the UMCG data according to the methods used in De Vette et al. [26], outlined in Appendix B.

### Reference logistic regression NTCP models

As a reference model for each toxicity, multivariable logistic regression models developed by Van den Bosch et al. [27] were used. The features included in each model, which are described in Appendix C, include clinical variables (such as the patient’s age or the location of the tumour) and mean doses to OARs. The weights of the logistic regression models were updated by refitting them using the development cohort of the current study, making them competitive comparators for the DL models.

### Data pre-processing and augmentation

**Fig. 1.**
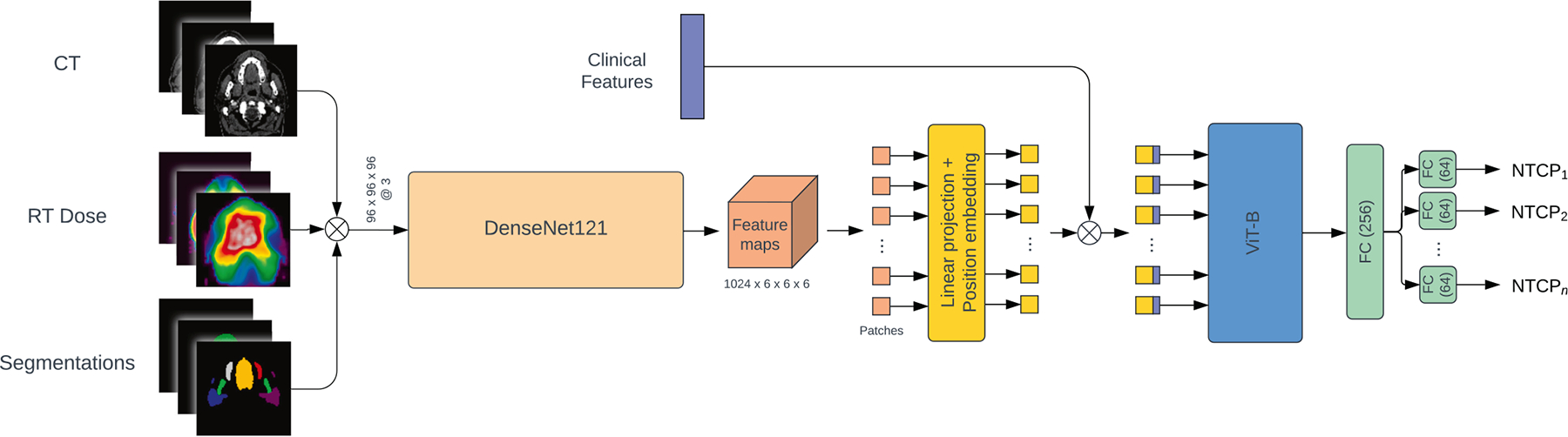
Diagram of the proposed multi-toxicity model, which utilises the TransRP architecture. Abbreviations: ViT = vision transformer, FC = fully connected layers.

To ensure consistency of the DL model inputs, the CT, OAR contours, planning dose distribution and clinical features are pre-processed using the same methodology as Chu et al. [10], with the exception of the voxel spacing, which we resampled to a resolution of 2 × 1 × 1 mm^3^ (height, depth, width), whereas 2 × 2 × 2 mm^3^ was previously used. The resulting CT, OAR contours and dose distribution are then stacked together as an image with three channels (3 × 96 × 192 × 192) when used as the input of the DL models (Fig. 1).

To improve the generalisability of the DL model, the size and diversity of the training data was enhanced using data augmentation techniques. First, a set of standard random transformations were applied to the 3D data, and then MixUp [28] was applied to improve the DL models’ calibration. These data augmentation steps are outlined in greater detail in Appendix A3.

### Deep learning architecture

This study employed an adaptation of the TransRP model architecture [29], which was previously shown to perform well in multi-outcome prediction for HNC patients [30]. As depicted in Fig. 1, the model consists of a 3D DenseNet121, followed by a Vision Transformer (ViT) and a fully connected network (FCN). The DenseNet121 is used to extract image features from the spatially-aligned 3D CT scans, dose distributions, and OAR segmentations. These features are then combined with clinical data (patient age, sex, and the pre-treatment toxicity score of all five toxicities) using the ViT to capture global-context interactions. The FCN, featuring toxicity-specific output heads, generates the final predictions for each toxicity simultaneously. We refer to this model as the multi-toxicity (MT) model. A detailed description of the model architecture is provided in Appendix D1. For comparison, DL models predicting single toxicities were also trained, resulting in a set of five single-toxicity (ST) models. They differ only from the MT model by the number of output heads (one rather than five) and the clinical features used (i.e. the pre-treatment scores of other toxicities were not included in each ST model).

All models were trained using 5-fold cross-validation on the development cohort. For internal validation, an ensemble approach was applied, averaging predictions across the five folds. Hyperparameter optimization was conducted using the Optuna library, with the mean validation loss across the five folds serving as the objective function. Further details on the training procedure and hyperparameter optimization can be found in Appendices D2 and D3.

### Evaluation metrics

The area under the receiver operating characteristic curve (AUC) was used as the primary evaluation metric of model performance. Furthermore, the overall model fit and calibration was evaluated using the Nagelkerke R^2^ and the adaptive calibration error (ACE) [31] metrics, and visually assessed using calibration curve plots. Missing endpoints are excluded from the calculations of the evaluation metrics. The results on the development, independent validation and external validation cohorts are reported.

### Leave-one-modality-out sub-analysis

To assess the contribution of each input modality (CT, dose, OAR contours, and clinical variables) we conducted a leave-one-modality-out (LOMO) analysis by retraining the MT model while omitting each modality individually.

### Endpoint combinations sub-analysis

Additionally, we investigated the effect of different toxicity combinations on model performance by training a series of dual-toxicity (DT) models; one for each of the possible pairwise combinations of the five toxicities used in the MT model. Each DT model followed the same training protocol as the ST models.

## Results

**Fig. 2.**
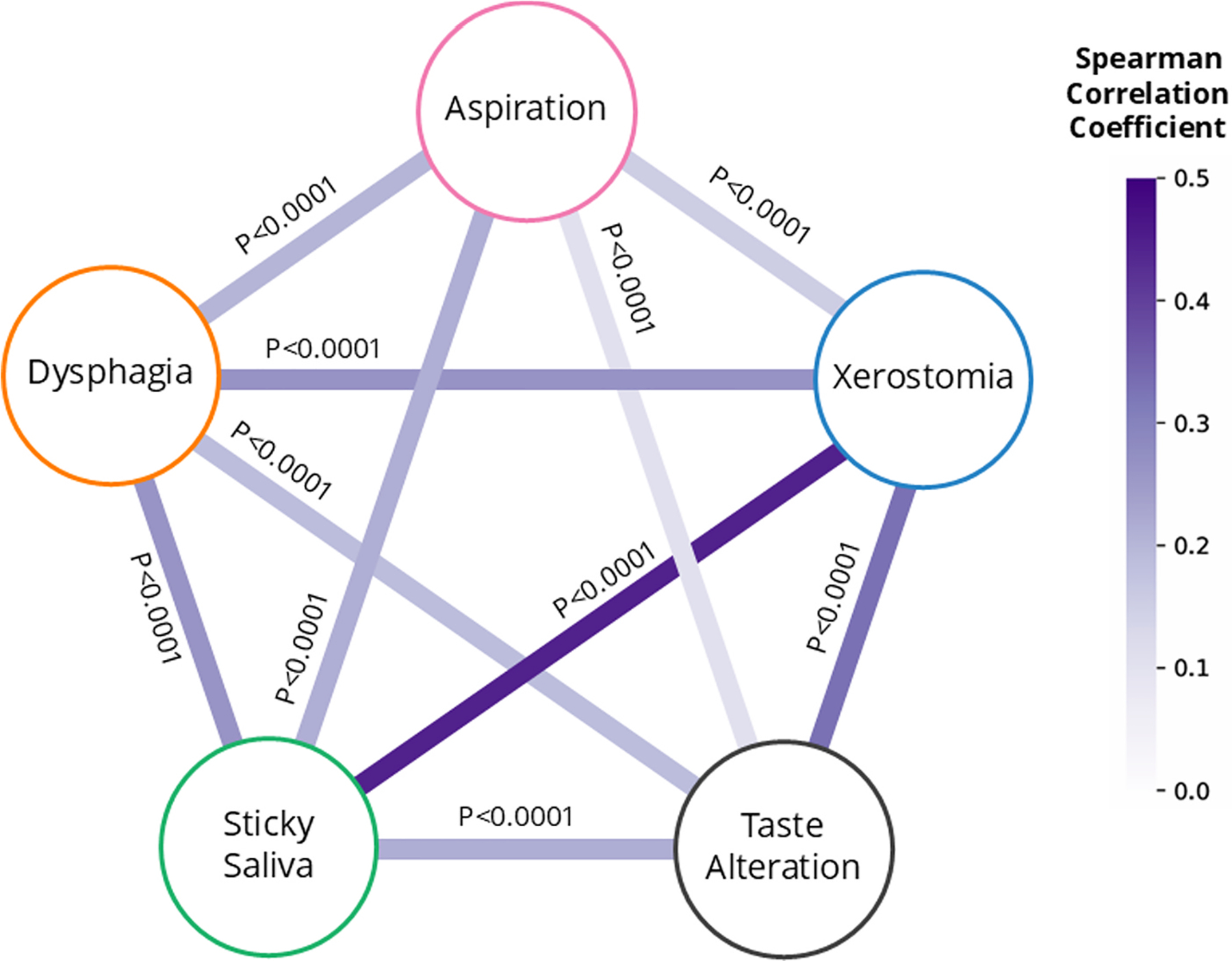
Spearman correlation coefficients between each of the pairs of the five (dichotomised) toxicity endpoints within the UMCG cohort. For each pair, patients missing either, or both, of the two 6-month toxicity ratings were excluded from the calculation of the correlation coefficient.

The UMCG cohort included 1,090 HNC patients (Appendix A1), which were split into a development cohort (n = 872) and an independent validation cohort (n = 218). The external validation cohort included 328 MDACC patients. There were no differences in patient characteristics between the development and independent validation cohorts. However, there was a significant difference between the UMCG and MDACC cohorts (Table 1). The spearman correlations between each pair of the five (dichotomised) toxicity endpoints within the entire UMCG cohort is presented in Fig. 2. The number of missing endpoints per patient within each cohort are indicated in Appendix E.

The AUCs for the reference, ST, and MT models were evaluated across the development, independent validation, and external validation cohorts for each toxicity separately (Table 2). In the development cohort, all models performed similarly, with AUC differences within each toxicity not exceeding 0.03. In the independent validation cohort, larger variations emerged; the MT model achieved a higher AUC on aspiration than the ST model (0.71) but performed as well as the reference model (both 0.74). For dysphagia and xerostomia, MT obtained the highest AUCs (0.83 and 0.80), surpassing both the ST (0.82 and 0.78) and reference (0.81 and 0.75) models on these endpoints. Conversely, on sticky saliva and taste alteration, the single-toxicity models both scored higher AUCs (0.71 for both endpoints) than the MT model (0.69 and 0.67). The difference in AUCs between the CITOR model compared to both the ST (DeLong test p = 0.047) and MT model (p = 0.012) for xerostomia were statistically significant, all other comparisons between models on the independent validation set were not (p > 0.05).

In the external validation cohort, the MT model achieved the highest AUCs for dysphagia (0.71) and sticky saliva (0.66) out of all models but performed worse on xerostomia (AUC = 0.58) than the other two models (both 0.60). All models, including the conventional NTCP models, showed lower AUCs in external validation cohort than in the independent validation cohort. Differences in AUCs within this cohort were generally not significant (p > 0.05), with the exception of the MT model compared to the ST model on sticky saliva (p = 0.028).

The R^2^ indicates that the MT model had the best goodness-of-fit of all models on taste alteration and sticky saliva in the independent validation cohort, while the ST model had the best fit on the remaining three toxicities. All models had a lower R^2^ on the external validation cohort compared to the development and independent validation cohorts. The ACE score generally did not differ within each endpoint by more than 0.02 between the models, with the exception of sticky saliva where the MT model showed the best calibration (0.06). Calibration plots (Appendix F) showed minor inter-model differences within each toxicity on each cohort.

Table 3 summarizes the impact of excluding each input modality on the MT model’s performance. Excluding the dose distribution led to the lowest AUC for dysphagia (0.78), taste alteration (0.59), and xerostomia (0.73). In contrast, the largest performance drops were observed for aspiration (0.60) and sticky saliva (0.61) when clinical features were removed. A slight increase in AUC was observed for dysphagia (0.84) and taste (0.71) when the model was trained without clinical features, and for sticky saliva (0.70) when the OAR contours were excluded from the model.

**Fig. 3.**
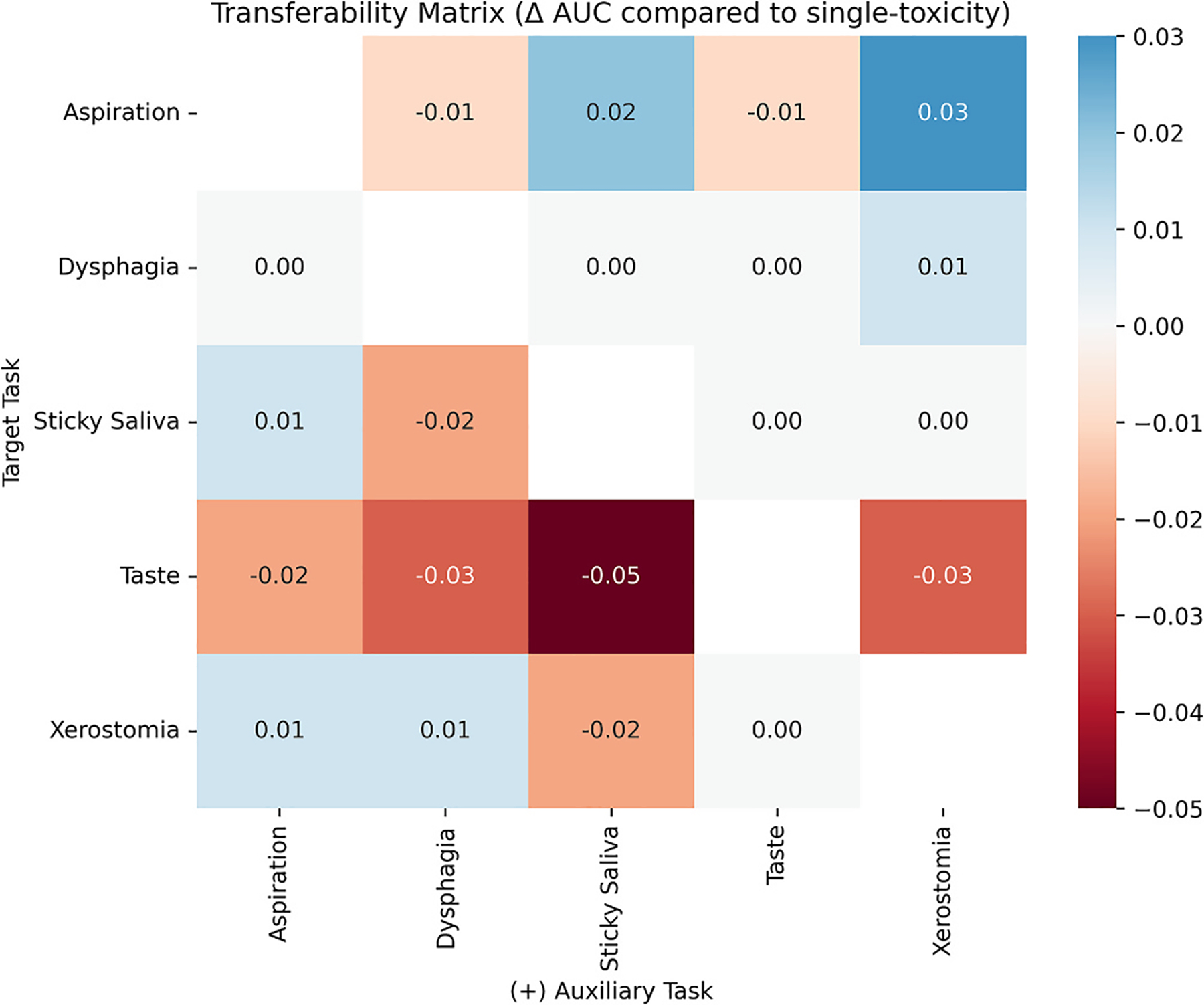
Transferability matrix showing the change in AUC (Δ AUC) for each target toxicity when paired with an auxiliary task in a dual-toxicity (DT) model, compared to the corresponding single-toxicity (ST) model. Rows represent the target toxicity, while columns represent the auxiliary toxicity. Positive values (blue) indicate improved performance when the auxiliary task is added, while negative values (red) indicate performance degradation.

Fig. 3 presents a transferability matrix showing the change in AUC when an auxiliary toxicity is added to a model predicting a target toxicity. Each cell indicates the AUC difference between dual- and single-toxicity models for the target task. Positive values (blue) reflect improved prediction; negative values (red) indicate degradation. Taste alteration consistently showed reduced performance when paired with other endpoints, particularly with sticky saliva (−0.05 AUC). Aspiration prediction improved most when paired with xerostomia (+0.03) and sticky saliva (+0.02). While most effects were limited, some pairs (aspiration–xerostomia, dysphagia–xerostomia, and aspiration–sticky saliva) showed mutual performance gains. Only aspiration and taste alteration showed mutual degradation.

Pairwise scatter plots of all models’ predictions on the independent and external validation cohorts (Appendix G) show that the MT model’s predictions across different toxicities are more strongly related than those of the ST or reference models, with predicted probabilities for each pair of toxicities fitting more closely to a trendline.

## Discussion

Our multi-toxicity (MT) deep learning (DL) NTCP model simultaneously predicts several radiotherapy-related toxicities in HNC patients, integrating 3D CT data, dose distributions, OAR contours, and clinical features. Overall, the MT model demonstrated performance comparable to both the single-toxicity (ST) DL models and reference NTCP models in terms of discrimination and calibration, while producing one-step prediction of all toxicity risks. Compared to the conventional approach, adopting a multi-toxicity modelling method led to slight improvements in aspiration, dysphagia, and xerostomia prediction. However, improvement was not uniform across all toxicities included in the MT model, as performance for sticky saliva and taste alteration declined compared to both the ST and reference models.

Our proposed MT model extends traditional single-toxicity NTCP methods by explicitly modelling relationships between toxicity endpoints within a unified DL framework. While many single-toxicity models have been developed to predict individual radiation-induced toxicities [32–34], multi-label DL architectures have shown efficacy in using DL models capturing complex relationships across correlated clinical outcomes using medical imaging as input [35–37]. Outside of DL, the recent multiomic Bayesian network approach by Nair et al. [38], which simultaneously predicts radiation pneumonitis (RP) and radiation esophagitis (RE) in NSCLC patients, further validates the potential for integrated NTCP modelling. Building on these concepts, our study demonstrates that MT models using DL-based approaches are technically feasible and raises an innovative hypothesis regarding heterogeneous inter-toxicity relationships, and are potentially more informative, pragmatic and actionable in instances where several distinct traditional single-toxicity-specific models would be required.

Beyond predictive performance, the clinical utility of the MT model lies in its potential to provide a comprehensive risk profile across multiple toxicity domains from a single model. In clinical practice, treatment decisions for HNC patients frequently involve navigating trade-offs between multiple toxicity risks — for instance, balancing the risk of dysphagia against xerostomia when optimising dose distributions. While the improvement in discrimination over single-toxicity models was modest, the MT framework’s value may therefore be better measured by its one-step holistic characterisation of patient risk and its practical feasibility than by AUC gains alone.

Compared to the ST and reference models, the MT model produced more strongly correlated predictions across toxicity endpoints (Appendix G), consistent with clinically observed co-occurrence of toxicities. Furthermore, the LOMO analysis focused on the influence of the model input modality; dysphagia, taste alteration, and xerostomia were all most dependent on dose distribution, while aspiration and sticky saliva relied more on clinical features. In some cases, adding clinical inputs reduced performance (e.g. taste alteration), indicating that there are both distinct, as well as shared, feature sets between the toxicities. This reflects similar patterns observed by Nair et al., who showed that their Bayesian network identified both distinct predictors for RP and RE and shared cross-organ features, including dependencies between lung (RP) oesophageal (RE) and radiomics features, highlighting interdependencies across organs. These findings explain why MT learning improved predictions for dose-dependent toxicities (e.g., dysphagia, xerostomia) but underperformed for endpoints dominated by unique features (e.g., taste alteration). In such cases, the model appeared to overestimate dependencies between toxicities, likely due to an over-reliance on shared anatomical, dose, or clinical features, as reflected by the discrepancy between predicted probabilities (Appendix G) and actual correlations (Fig. 2). This risks reducing specificity, making it more difficult to distinguish patients at risk for a specific toxicity from those indiscriminately predicted to experience multiple toxicities, and underscores a key challenge in multi-toxicity modelling: avoiding the over-estimation of true clinical co-occurrence patterns.

Analysis of different endpoint combinations (Fig. 3) showed that adding auxiliary toxicities had mixed effects on performance in predicting other toxicities. For example, performance in dysphagia prediction was largely unaffected by the addition of auxiliary toxicities within the model. Despite the known link between taste and salivary dysfunction [39,40], pairing taste alteration with xerostomia or sticky saliva consistently reduced performance, suggesting this endpoint is poorly suited to multi-toxicity modelling. Yet, the presence of mutually-positive pairs suggests that certain combinations of toxicities do benefit from adopting a multi-toxicity modelling approach.

Although some patients in the development cohort had missing toxicity labels, this did not appear to impair learning of inter-toxicity relationships. Missingness was limited (8.8% of all 5 × 872 possible labels, see Appendix E) and largely systematic, mainly affecting physician-rated dysphagia or all four patient-reported endpoints. Moreover, retraining the model on different endpoint combinations or on patients with fewer missing labels did not meaningfully improve performance. Together, these findings suggest that including patients with partially incomplete endpoint data did not adversely affect modelling of inter-toxicity interactions.

A limitation of this study was the size of the development cohort, which may have been insufficient to fully support the complexity of the multi-toxicity modelling task. Deep learning models generally require large, high-quality datasets—an ongoing challenge in medical imaging—and this requirement is amplified when optimizing for multiple, interrelated endpoints simultaneously rather than for a single, well-defined outcome. An additional limitation is that there were clinically-relevant differences between the UMCG and MDACC cohorts in both patient characteristics (e.g. all MDACC patients where oropharynx patients), fractionation schemes, and endpoint assessment rating scale. This may explain why all of the models (including the reference models) exhibited limited generalisability to the external data. Variations in OAR delineation methods may also have contributed to this limited generalisability, although we believe that this effect is minimal as de Vette et al. [26] showed that atlas-based and DL-based contours yielded similar mean dose values. Another potential limitation of this study was that the endpoints were predominantly patient-reported (except for physician-rated dysphagia). While patient ratings provide valuable insight into toxicity burdens, their variability may obscure the inter-toxicity mechanisms that the model aims to learn. Physician-rated or functional measures could offer an alternative, but are likewise limited by inconsistencies in implementation and interpretation—such as variation in salivary flow assessment [41,42] and clinician grading [43]. We also did not explicitly study the use of (class-wise) loss corrections or reweighting during model training, while a variety of loss-based and feature-based methods to adapt to and correct co-occurrence and correlations of labels already exist [44]. Given the overestimation of toxicity correlations that we ultimately found in our results, future work should carefully assess prediction dependencies against empirical toxicity relationships and consider loss-based interventions for when necessary, or explore alternative model architecture strategies. Future works may also look to integrate the multi-toxicity framework with dynamic modelling strategies capable of capturing temporal evolution in toxicity risk, which could enable serial, longitudinal risk predictions and ultimately support more adaptive, patient-centred follow-up strategies.

## Conclusion

A DL NTCP model predicting multiple toxicities simultaneously was developed and validated on two large HNC patient cohorts. This multi-toxicity DL model outperformed the conventional NTCP and single-toxicity DL models for aspiration, dysphagia, and xerostomia prediction on the independent validation cohort. Herewith, this study demonstrates the feasibility and promise of adopting a more holistic deep learning-based modelling approach that considers the interactions between multiple radiation-induced late-stage toxicities.

## Supplementary Material

Appendix A. Supplementary data

## Figures and Tables

**Table 1:** Patient characteristics.

	Development Cohort		Independent Validation Cohort		External Validation Cohort		p-values UMCG-MDACC cohort
Total	872		218		328		
**Sex** (%)							<0.001^†^
Female	213	(24)	50	(23)	35	(11)	
Male	659	(76)	168	(77)	293	(89)	
**Age** (mean (SD))	64	(10)	64	(10)	60	(9)	<0.001^‡^
**Tumour site** (%)							<0.001^†^
Oropharynx	321	(37)	82	(38)	328	(100)	
Nasopharynx	37	(4)	9	(4)	0	(0)	
Hypopharynx	75	(9)	19	(9)	0	(0)	
Larynx	381	(44)	96	(44)	0	(0)	
Oral Cavity	47	(5)	12	(5)	0	(0)	
Other	11	(1)	0	(0)	0	(0)	
**T-stage** (%)							<0.001^†^
T0	2	(0)	0	(0)	5	(2)	
Tis	4	(0)	2	(1)	0	(0)	
T1	152	(17)	42	(19)	100	(30)	
T2	263	(30)	44	(20)	127	(39)	
T3	207	(24)	72	(33)	48	(15)	
T4	263	(28)	58	(27)	47	(14)	
Tx	1	(0)	0	(0)	1	(0)	
**N-stage** (%)							<0.001^†^
N0	409	(47)	100	(46)	23	(7)	
N1	81	(9)	20	(9)	117	(36)	
N2	356	(41)	91	(42)	181	(55)	
N3	26	(3)	7	(3)	6	(2)	
Unknown	0	(0)	0	(0)	1	(0)	
**Smoking** (%)							<0.001^†^
Current smokers	414	(47)	101	(46)	0	(0)	
Past smokers	348	(30)	92	(42)	0	(0)	
Never smokers	110	(13)	25	(12)	0	(0)	
Unknown	0	(0)	0	(0)	359	(100)	
**OPC P16 HPV** (%)							<0.001^†^
Positive	143	(17)	32	(15)	13	(4)	
Negative	151	(17)	41	(19)	239	(73)	
Unknown	27	(3)	9	(4)	76	(23)	
Other tumour sites	551	(63)	136	(62)	0	(0)	
**WHO** (%)							<0.001^†^
0	612	(70)	156	(72)	0	(0)	
1	216	(25)	53	(24)	0	(0)	
2	41	(5)	9	(4)	0	(0)	
3	2	(0)	0	(0)	0	(0)	
4	1	(0)	0	(0)	0	(0)	
Unknown	0	(0)	0	(0)	328	(100)	
**Treatment technique** (%)							<0.001^†^
IMRT	389	(45)	90	(41)	60	(18)	
VMAT	296	(34)	80	(37)	189	(58)	
IMPT	126	(14)	30	(14)	59	(18)	
3D-CRT	56	(6)	13	(6)	0	(0)	
IMRT + VMAT	6	(1)	5	(2)	20	(6)	
**Systemic treatment** (%)							<0.001^†^
With	340	(39)	101	(46)	253	(77)	
Without	532	(61)	117	(54)	70	(23)	
**CT with contrast** (%)							<0.001^†^
No	151	(17)	37	(17)	328	(100)	
Yes	721	(83)	181	(83)	0	(0)	
**CT with metal artefact** (%)							<0.001^†^
None	450	(52)	109	(50)	51	(16)	
Little/Medium	191	(22)	56	(26)	143	(44)	
Heavy	231	(26)	53	(24)	134	(41)	
**Baseline aspiration** (%)							1.0^†^
None	718	(82)	177	(81)	272	(83)	
Little	121	(14)	35	(16)	44	(13)	
Moderate-to-severe	33	(4)	6	(3)	12	(4)	
**Aspiration at 6 months** (%)							0.345^†^
None-to-little (non-event)	721	(83)	175	(80)	277	(84)	
Moderate-to-severe (event)	59	(7)	19	(9)	16	(5)	
Unknown	92	(10)	24	(11)	35	(11)	
**Baseline dysphagia** (%)							<0.001^†^
Grades 0 to 1	686	(79)	182	(84)	292	(89)	
Grade 2	119	(14)	20	(9)	13	(4)	
Grades 3 to 4	67	(7)	16	(7)	23	(7)	
**Dysphagia at 6 months** (%)							0.961^†^
Grades 0 to 1 (non-event)	634	(73)	160	(73)	237	(73)	
Grades 2 to 4 (event)	210	(24)	48	(22)	80	(24)	
Unknown	28	(3)	10	(5)	11	(3)	
**Baseline sticky saliva** (%)							1.0^†^
None	575	(66)	139	(64)	250	(76)	
Little	209	(24)	55	(25)	44	(14)	
Moderate-to-severe	88	(10)	24	(11)	34	(10)	
**Sticky saliva at 6 months** (%)							0.005^†^
None-to-little (non-event)	523	(60)	136	(62)	226	(69)	
Moderate-to-severe (event)	260	(30)	61	(28)	67	(20)	
Unknown	89	(10)	21	(10)	35	(11)	
**Baseline taste alteration** (%)							0.338^†^
None	695	(80)	176	(81)	273	(83)	
Little	131	(15)	35	(16)	34	(10)	
Moderate-to-severe	46	(5)	7	(3)	21	(4)	
**Taste alteration at 6 months** (%)							<0.001^†^
None-to-little (non-event)	576	(66)	142	(65)	177	(54)	
Moderate-to-severe (event)	202	(23)	52	(23)	114	(35)	
Unknown	94	(11)	24	(11)	37	(11)	
**Baseline xerostomia** (%)							<0.001^†^
None	502	(58)	120	(55)	240	(73)	
Little	280	(32)	79	(36)	45	(14)	
Moderate-to-severe	90	(10)	19	(9)	43	(13)	
**Xerostomia at 6 months** (%)							<0.001^†^
None-to-little (non-event)	453	(52)	104	(47)	165	(50)	
Moderate-to-severe (event)	334	(38)	93	(43)	128	(39)	
Unknown	85	(10)	21	(10)	35	(11)	

‡ Kruskal-Wallis test.

† Chi-squared test. TNM staging version 7 for the development and independent validation cohorts, TNM staging version 7 (n = 201) and 8 (n = 126) for external test cohort.

**Table 2:** Model performances.

		Development Cohort			Independent Validation Cohort			External Validation Cohort		
		Reference	ST	MT	Reference	ST	MT	Reference	ST	MT
AUC [95% CI]	Aspiration	**0.74** [0.59–0.88]	0.71 [0.55–0.85]	0.72 [0.56–0.86]	**0.74** [0.61–0.86]	0.71 [0.57–0.83]	**0.74** [0.61–0.85]	**0.72** [0.61–0.83]	0.68 [0.54–0.81]	0.68 [0.52–0.81]
	Dysphagia	0.80 [0.72–0.87]	**0.82** [0.75–0.89]	**0.82** [0.75–0.89]	0.81 [0.73–0.87]	0.82 [0.74–0.88]	**0.83** [0.76–0.89]	0.67 [0.60–0.73]	0.66 [0.58–0.72]	**0.71** [0.64–0.77]
	Sticky saliva	**0.71** [0.61–0.78]	**0.71** [0.62–0.79]	0.68 [0.58–0.76]	**0.72** [0.62–0.79]	0.71 [0.63–0.79]	0.69 [0.61–0.77]	0.63 [0.55–0.70]	0.60 [0.52–0.68]	**0.66** [0.58–0.73]
	Taste	0.71 [0.61–0.80]	**0.73** [0.64–0.82]	0.72 [0.64–0.81]	**0.71** [0.62–0.78]	**0.71** [0.63–0.78]	0.67 [0.60–0.75]	0.55 [0.48–0.61]	**0.59** [0.52–0.65]	0.58 [0.51–0.64]
	Xerostomia	**0.75** [0.69–0.84]	0.74 [0.66–0.82]	0.72 [0.64–0.80]	0.75 [0.70–0.83]	0.78 [0.72–0.85]	**0.80** [0.74–0.86]	**0.60** [0.53–0.66]	**0.60** [0.53–0.66]	0.58 [0.52–0.65]
ACE	Aspiration	0.05	0.05	0.05	0.05	0.06	0.04	0.04	0.04	0.05
	Dysphagia	0.07	0.07	0.07	0.05	0.07	0.08	0.08	0.06	0.06
	Sticky saliva	0.08	0.09	0.07	0.09	0.12	0.06	0.13	0.13	0.11
	Taste	0.08	0.08	0.08	0.07	0.09	0.07	0.11	0.09	0.09
	Xerostomia	0.09	0.10	0.10	0.11	0.11	0.12	0.11	0.14	0.12
R^2^	Aspiration	0.04	0.08	0.03	0.10	0.04	0.08	0.05	0.03	−0.02
	Dysphagia	0.22	0.23	0.24	0.26	0.24	0.26	0.07	0.10	0.13
	Sticky saliva	0.09	0.11	0.08	0.14	0.10	0.09	−0.04	−0.07	−0.01
	Taste	0.09	0.14	0.13	0.08	0.09	0.07	−0.04	0.02	−0.02
	Xerostomia	0.18	0.17	0.14	0.21	0.17	0.19	0.01	−0.01	−0.04

Bold is highest AUC per toxicity on each cohort. Results on the validation cohorts are of the ensemble of models, while the development set is the average across all five folds of K-fold cross validation. Abbreviations: ST = single-toxicity DL NTCP model, MT = multi-toxicity DL NTCP model, CI = confidence interval.

**Table 3:** Results of LOMO analysis.

	Aspiration	Dysphagia	Sticky Saliva	Taste	Xerostomia
All	**0.74**	0.83	0.69	0.67	**0.80**
No CT	0.72	0.83	0.68	0.66	0.78
No 3D dose distribution	0.70	0.78	0.68	0.59	0.73
No OAR contours	0.71	0.83	**0.70**	0.66	**0.80**
No clinical features	0.60	**0.84**	0.61	**0.71**	0.77

Bold is highest AUC per toxicity, while underlined is lowest.

## Data Availability

In accordance with the *Final NIH Policy for Data Management and Sharing* NOT-OD-21-013, U.S.-derived data that support the findings of this study are openly available in an NIH-supported generalist scientific data repository (figshare) at 10.6084/m9.figshare.30710006 no later than the time of an associated publication.

## Appendix A. Supplementary data

Supplementary data to this article can be found online at https://doi.org/10.1016/j.radonc.2026.111486.

## Abbreviations:

ACE: adaptive calibration error
AUC: Area under the curve
CTCAE: Common Terminology Criteria for Adverse Events
DL: Deep learning
DT: dual-toxicity model
EORTC QLQ-H&N35: European Organisation for Research and Treatment of Cancer Quality of Life Questionnaire Head and Neck Module
HNC: Head and neck cancer
LOMO: leave-one-modality-out
MDACC: MD Anderson Cancer Centre
MDASI-HN: MD Anderson Symptom Inventory-Head and Neck Module
MT: multi-toxicity model
NSCLC: non-small cell lung cancer
NTCP: Normal tissue complication probability
OAR: Organ at risk
PSS-HN: Performance Status Scale for Head and Neck cancer
RE: radiation esophagitis
RP: radiation pneumonitis
ST: single-toxicity model
UMCG: University Medical Centre Groningen
