# Supplementary material for: A multi-toxicity deep learning approach for normal tissue complication probability modelling in head and neck cancer patients receiving radiotherapy
Source: Radiother Oncol. Author manuscript; Available in PMC 2026 Apr 2. (PMC13039088; doi:10.1016/j.radonc.2026.111486)
Supplement: Appendix A. Supplementary data [file NIHMS2160166-supplement-Appendix_A__Supplementary_data.docx]

# Appendix A: Data

## A1: Data exclusion diagrams


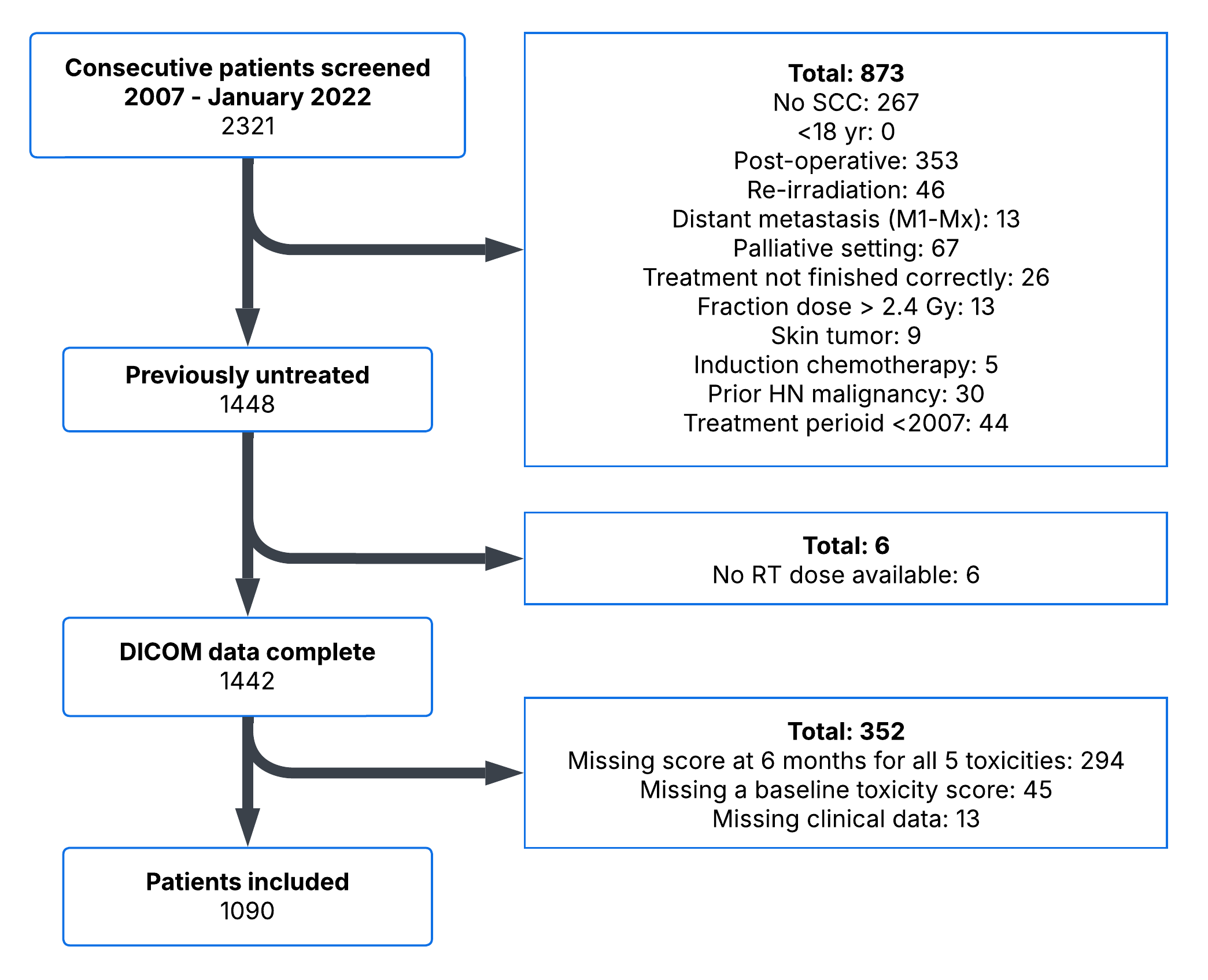


Figure A.1: Exclusions for the UMCG cohort.


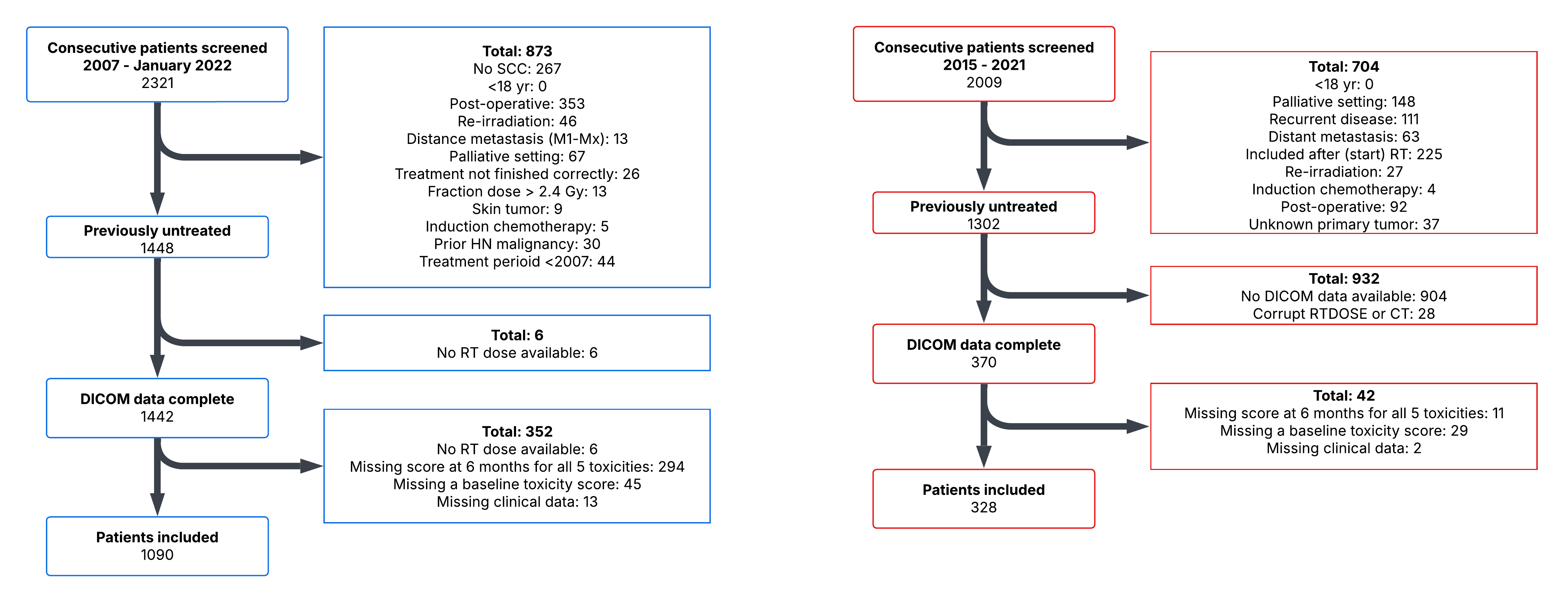


Figure A.2: Exclusions for the MDACC cohort.

## A2: Number of missing organ-at-risk contours

Table A.1 outlines how many OAR contours were missing from the UMCG cohort (manually delineated) and the MDACC cohort (atlas-based auto-contouring using the Elekta ADMIRE algorithm). These missing OAR contours were instead obtained by using the deep learning contour (DLC) algorithm by Van Dijk et al. [1].

| Organ | Number of DLC contours | |
| --- | --- | --- |
|  | UMCG cohort (N=1090) | MDACC cohort (N=328) |
| Buccal mucosa left | 16 | 328 |
| Buccal mucosa right | 17 | 328 |
| Cricopharyngeal muscle | 15 | 14 |
| Oesophagus | 19 | 21 |
| Glottic area | 25 | 20 |
| Mandible | 33 | 7 |
| Oral cavity | 16 | 8 |
| Parotid gland left | 14 | 2 |
| Parotid gland right | 15 | 2 |
| PCM inferior | 15 | 16 |
| PCM medium | 15 | 18 |
| PCM superior | 15 | 15 |
| Submandibular gland left | 16 | 9 |
| Submandibular gland right | 17 | 7 |
| Supraglottic larynx | 17 | 15 |
| Thyroid | 25 | 214 |

Table A.1: Number of OAR contours within the UMCG and MDACC cohorts that were obtained using a deep learning contouring (DLC) algorithm.

## A3: Data augmentation

Data augmentation was utilised to increase the size and diversity of the training dataset, in order to improve the DL models’ generalisation capabilities. This augmentation was applied on each epoch in two phases. First, for each patient a set of random 3D transformations were applied to the 3D inputs (i.e. the CT, OAR segmentations, and the dose distribution). Each of the following random transformations were applied independently with a probability of 0.5, using the MONAI library [2]:

- Random cropping to a dimension of $96\times192\times192$ (height, width, depth)
- Horizontal flipping (over the y-axis)
- Rotation within a range of [-15°, 15°]
- Adding Gaussian noise with a mean of 0 and std of 0.01
- Affine transformation (up to 6 voxels in each direction)

Then, the data is further augmented using the MixUp algorithm [3]. MixUp creates a synthetic training example $\left( \hat{x},\hat{y} \right)$ by

$$\hat{x}=\lambda x_{i}+\left( 1-\lambda\right)x_{j}, \mathrm{where}x_{i},x_{j} are input vectors$$

$$\hat{y}=\lambda y_{i}+\left( 1-\lambda\right)y_{j}, where y_{i},y_{j} are label vectors$$

where $\left( x_{i},y_{i} \right)$, $\left( x_{j},y_{j} \right)$ are two examples drawn at random from the training batch (and where $i \neq j$). $\lambda\in\left[ 0,1 \right]$ is a value sampled from a Beta distribution, defined by an alpha parameter $\left( \alpha,\alpha\right)$, for $\alpha\in\left( 0, \infty\right)$, which controls the strength of the mixing between two input-label pairs. During training, we repeat this process until enough synthetic examples have been generated to refill the batch size.

This mixing encourages the DL model to behave linearly in-between the training examples, improving the calibration and helping to mitigate any oscillations when predicting outside of the training examples.

In Figure A.3, we present an example of what a synthetic training input $\hat{x}$ created by MixUp looks like. There, from top to bottom, the CT, dose distribution, OAR segmentations and clinical features (tabular data) from two random samples $x_{i}$ and $x_{j}$ are mixed together according to a random value of $\lambda$, which in this case is 0.625.


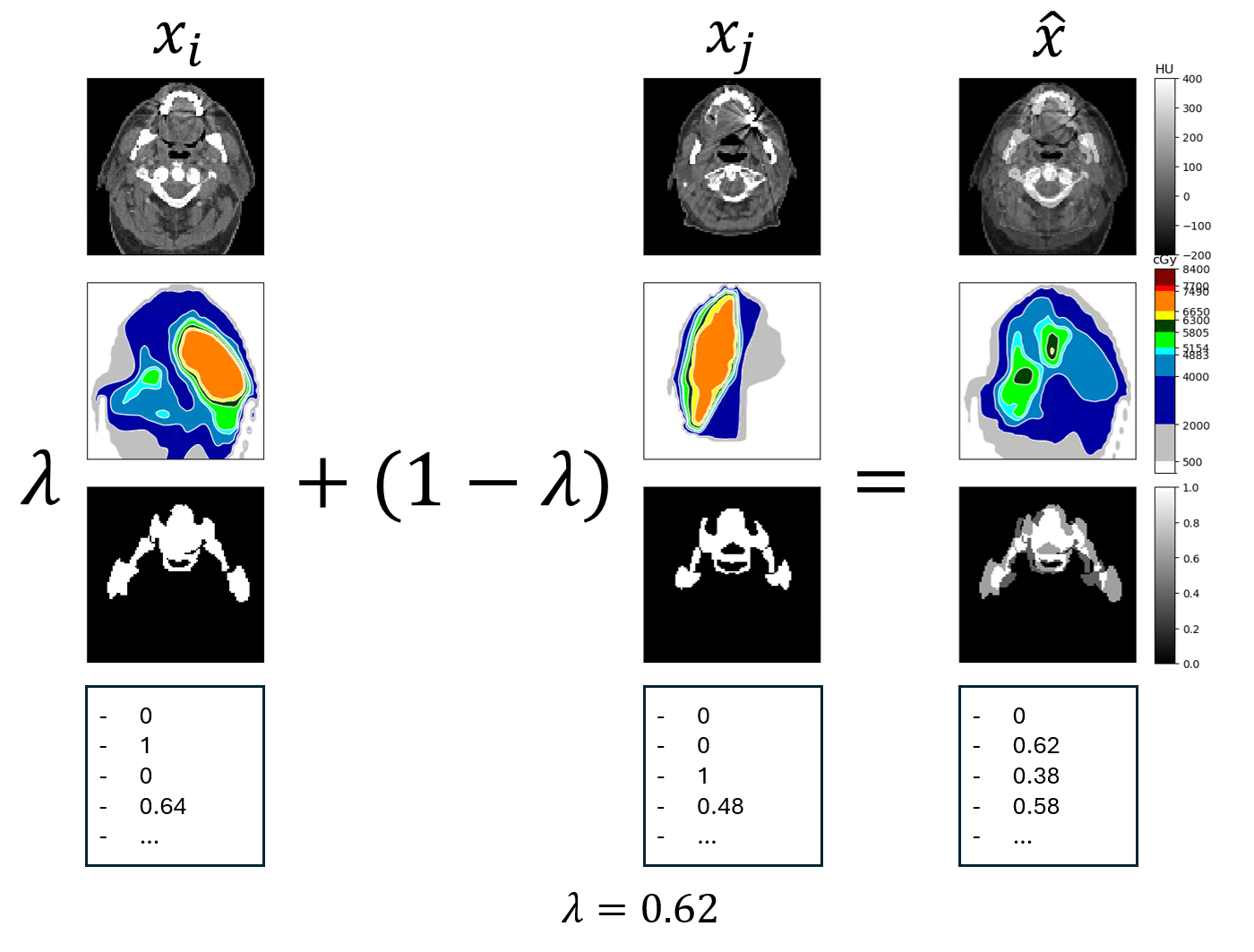


Figure A.3: Example of a synthetic datapoint generated by MixUp augmentation. See text for description.

# Appendix B: Conversion of Toxicity Rating Scales

The toxicity ratings for the MDACC cohort were obtained using different assessment methods than in the UMCG cohort. Dysphagia was assessed in the MDACC cohort using the PSS-HN “diet normalcy” rating (rated out of 100), while in the UMCG cohort dysphagia was assessed using the CTCAEv4.0 criteria [4]. The remaining toxicities were assessed using the MD Anderson Symptom Inventory-Head and Neck Module (MDASI-HN, rated on a scale of 0 to 10)[5] within the MDACC cohort, and the EORTC QLQ-H&N35 questionnaire [6] in the UMCG cohort.

To convert the MDACC ratings to the same scales as in the UMCG cohort, we applied the thresholds identified by De Vette et al. [7], where logistic regression models developed on a cohort of UMCG patients were externally validated on an MDACC cohort. The conversion scale is described in Table B.1.

|  | **MDACC rating** | **Conversion to UMCG rating scale** | | | | | |
| --- | --- | --- | --- | --- | --- | --- | --- |
|  | PSS-HN category | CTCAEv4.0 rating | | | | | |
|  |  | Grades 0-1 | | Grade 2 | | Grades 3-4 | |
| Dysphagia | ‘diet normalcy’ | >70 | | >50 | | ≤50 | |
|  |  |  | |  | |  | |
|  | MDASI-HN category | EORTC QLQ-H&N35 rating | | | | | |
|  |  | Not at all | A little | | Moderate | | Severe |
| Aspiration | ‘choking or coughing’ | <2 | <5 | | <8 | | ≥8 |
| Sticky Saliva | ‘mucus in the mouth or throat’ | <2 | <4 | | <7 | | ≥7 |
| Taste | ‘problems with tasting food’ | <2 | <4 | | <7 | | ≥7 |
| Xerostomia | ‘dry mouth’ | <2 | <4 | | <7 | | ≥7 |

Table B.1: *conversion scales to adapt the MDACC toxicity scores to the UMCG grading scales (CTCAEv4.0 and EORTC QLQ-H&N35).*

# Appendix C: Reference logistic regression NTCP models

The reference models were multivariable logistic regression NTCP models as in Van Den Bosch et al. [8] The normal tissue complication probabilities are calculated by each model as:

$$\text{NTCP}= \frac{1}{1+e^{-y}} ,$$

Where $y$ is defined by fitting the regression parameters $\beta$ for the $d$ input variables $x$:

$$y=\beta_{1}x_{1}+\beta_{2}x_{2}+ \ldots+\beta_{d}x_{d} + c$$

Where $c$ is the intercept. For the dysphagia, sticky saliva and xerostomia reference models, $y$ is obtained by taking the mean coefficients of two separately fitted submodels (with each not incorporating one of the two mean OAR dose features [9]). The features included in each reference model are shown in Table C.1.

| Model | Variables | **Original coefficients** | **Refitted coefficients** |
| --- | --- | --- | --- |
| Aspiration | Mean dose middle PCM  Baseline toxicity ‘a little’  Baseline toxicity ‘moderate-to-severe’ | 0.0339  1.1389  0.9472  -4.3542 (intercept) | 0.0183  1.4086  2.0869  -3.9003 (intercept) |
| Dysphagia | Mean dose oral cavity_1_  Mean dose superior PCM_2_  Mean dose middle PCM  Mean dose inferior PCM  Baseline toxicity grade 2  Baseline toxicity grades 3-4  Tumour site Pharynx  Tumour site Larynx | 0.0352  0.0276  0.0111  0.0156  1.0968  1.5068  -0.7349  -0.9027  -4.7065 (intercept) | 0.0203  0.0165  0.0109  0.0094  1.0003  1.1852  -0.7894  -1.2493  -3.0176 (intercept) |
| Sticky Saliva | Mean dose submandibular glands_1_  Sum of the square roots of the mean doses to each parotid gland_2_  Baseline toxicity ‘a little’  Baseline toxicity ‘moderate-to-severe’ | 0.0136  0.0684  0.5460  0.9414  -2.3496 (intercept) | 0.0120  0.0523  0.8034  1.5565  -2.1110 (intercept) |
| Taste | Mean dose oral cavity  Sum of the square roots of the mean doses to each parotid gland  Age | 0.1870  0.0872  0.3611  -4.5092 (intercept) | 0.3636  0.0876  0.0379  -5.7507 (intercept) |
| Xerostomia | Mean dose submandibular glands_1_  Sum of the square roots of the mean doses to each parotid gland_2_  Baseline toxicity ‘a little’  Baseline toxicity ‘moderate-to-severe’ | 0.0197  0.1074  0.5337  1.3017  -2.5497 (intercept) | 0.0213  0.1055  0.7238  1.4111  -2.5054 (intercept) |

Table C.1: *Original and refitted coefficients for each of the reference models. Subscripts ‘1’ and ‘2’ refer to features which are included in only one of two separately fitted sub-models (toxicities without this superscript did not utilise sub-models in the refitting process). Abbreviations: PCM = pharyngeal constrictor muscle. Age was normalised from 0-100 to 0-1.*

# Appendix D: DL Model and Hyperparameters

## D1. TransRP model architecture

The TransRP model, shown in Figure D.1, consists of three modules; first, a 3D DenseNet121 is used as an image feature extractor on the 3D CT, dose and OAR segmentation arrays, which are stacked together into an array of three channels (3, 96, 192, 192). The resulting feature maps are then passed onto a vision transformer (ViT). In order to provide the ViT with higher-resolution feature maps, such that localisation of features is still possible with a reasonable level of granularity, we adjust the DenseNet by removing the fourth, and final, dense block from the model. Thus, the DenseNet module is shallower than a standard DenseNet. The resulting feature maps have a dimension of (1024, 6, 12, 12) (as opposed to (1024, 3, 6, 6) if we were to keep the final dense block).

The second module is a ViT-based module. Here, the feature maps are divided into patches of size 1x1x1 (thus there are 864 patches), upon which linear projection, to a vector of length 256, and position embedding are applied. Then, the clinical features are inserted into the model by concatenating them to each of these position embedded vectors. This results in 864 vectors of length 256 + C, where C is the number of clinical features. These vectors are subsequently passed onto the ViT itself. The ViT itself then learns global-context features by modelling the interactions between all of the aforementioned patches (containing both the image features and clinical features) through the use of multi-head self-attention (MHSA). Here, we use a custom ViT with 4 transformer encoder blocks, each having 64 attention heads and a hidden linear layer of size 1024.


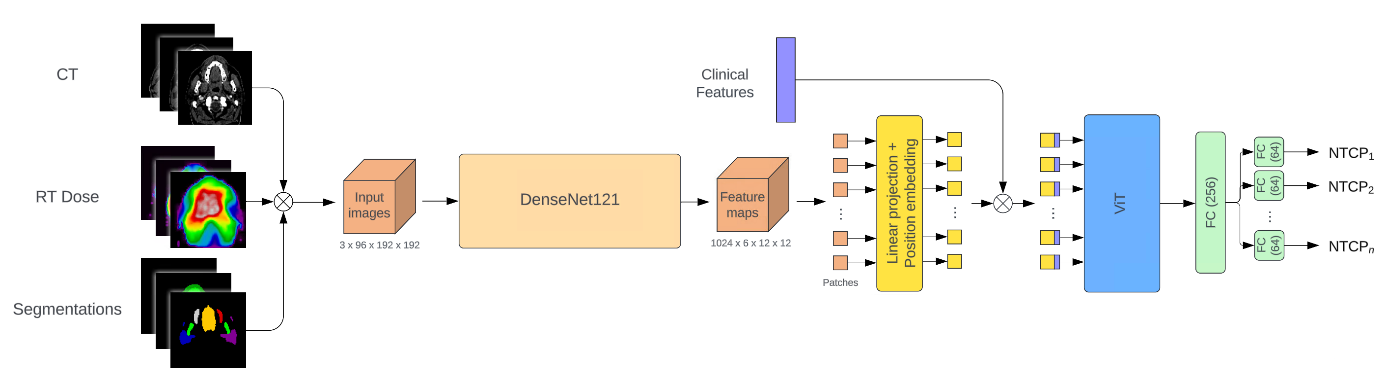


Figure D.1: schematic of the TransRP model architecture.

A feature vector is extracted from the ViT by taking the mean of the 864 resulting vectors, which is then passed to the final module; a fully connected network (FCN). We maintained this approach, as in the original TransRP implementation, as experiments using learnable classification tokens (also known as ‘CLS tokens’) [10] for each toxicity (i.e. 5 CLS tokens for the 5-endpoint MT model, and 1 for the 1-endpoint ST models) did not yield any change in performance within the current cohort. The FCN consists of one ‘shared’ linear layer (of size 256, with LReLU) and then five ‘non-shared’ linear layers (of size 64). These `non-shared` linear layers act as output heads for each toxicity, and each output a single value. Dropout is applied to both types of linear layers, while the sigmoid activation function is applied only to the output layer.

## D2. Training

All models were trained using 5-fold cross-validation on the internal development cohort. Each fold was created using stratified sampling, ensuring a balance of patients in each fold according to the radiotherapy treatment technique used, the location of the patient's tumour, whether their CT scan was taken with or without contrast, and the CT metal artefact rating.

All models were trained for either 100 epochs, or until the early stopping criteria was met, whichever came first. Early stopping of training was applied when the mean validation loss did not decrease beyond the lowest achieved value for ten consecutive epochs.

During model evaluation on each epoch, any missing endpoint data (i.e. missing labels) are masked out, and are thus excluded from the calculation of the evaluation metrics. The same applies to the loss on the validation set. However, in order to be able to apply the MixUp algorithm (see Appendix A), missing values within the training set are set to 0 (no toxicity), which enables the mixing of the label data on each epoch.

The data augmentation and model training was implemented using Python 3.11.3, PyTorch 2.2.0 and Monai 1.4.0 [2], [11], and the experiments were conducted using an Intel Xeon Platinum 8358 CPU, 80GB RAM, and an Nvidia A100 GPU with 40GB VRAM. The experiments were implemented in the PR3DICTR framework, the code for which is available on GitHub: <https://github.com/DLinRadiotherapyUMCG/PR3DICTR/>

## D3. Hyperparameter tuning

The model and training hyperparameters were optimized using the Optuna library [12]. In each experiment, 100 trials are run. Each trial consists of one 5-fold cross-validation run, where DL models are trained with a certain configuration of hyperparameters sampled from the search space. In the first 50 trials, the hyperparameters are selected randomly. This gives the Optuna algorithm a sufficient amount of samples for the remaining trials with which to further optimise the hyperparameters such that they minimised the objective, which was set to be the mean validation losses of each of the five toxicities of the model.

The search space of the hyperparameters which were optimised are shown in Table D.1, as is the optimal value of each hyperparameter in the final model.

| **Parameter** | **Options** | **Optimal** |
| --- | --- | --- |
| **TransRP Model Architecture** | | |
| DenseNet version | [121, 169, 201] | 121 |
| DenseNet kernel size of first conv. layer | [3,5,7] | 5 |
| ViT embedding dimension | [128, 256, 512, 768] | 256 |
| ViT n layers | [2, 4, 6, 8] | 4 |
| ViT n heads | [16, 32, 64, 128] | 64 |
| ViT MLP dimension | [256, 512, 1024] | 1024 |
| ViT dropout probability | 0 to 0.3 | 0 |
| n shared linear layers | [0, 1, 2, 3] | 1 (size=256) |
| n non-shared linear layers | [0, 1, 2, 3] | 1 (size=64) |
| linear layer sizes | [8, 16, 32, 64, 128, 256, 512] | See above |
| LReLU alpha | 0 to 0.1 | 0.07 |
| dropout probability | 0 to 0.3 | 0.03 |
| **Training** | | |
| Batch size | [2, 4, 8, 16] | 8 |
| Learning rate | [1e-5, 5e-5, 1e-4, 5e-4, 1e-3] | 1e-4 |
| Optimiser | [Adam, AdamW, AdaBound, SGD] | Adam |
| Weight decay | [0, 0.01, 0.05] | 0 |
| MixUp alpha | 0 to 5 | 1.7 |
| Apply gaussian noise image augmentation (mean=0, std=0.01) | [True, False] | True |

Table D.1: Hyperparameter search space and optimal values.

# Appendix E: Endpoint data missingness

| Number of missing endpoint labels | Development cohort | Independent validation cohort | External validation cohort |
| --- | --- | --- | --- |
| 0 | 738 | 185 | 280 |
| 1 | 46 | 9 | 13 |
| 2 | 4 | 2 | 0 |
| 3 | 2 | 1 | 0 |
| 4 | 82 | 21 | 35 |

Table E1: The number of missing 6-month toxicity endpoints per patient, in each cohort.

# Appendix F: Calibration plots


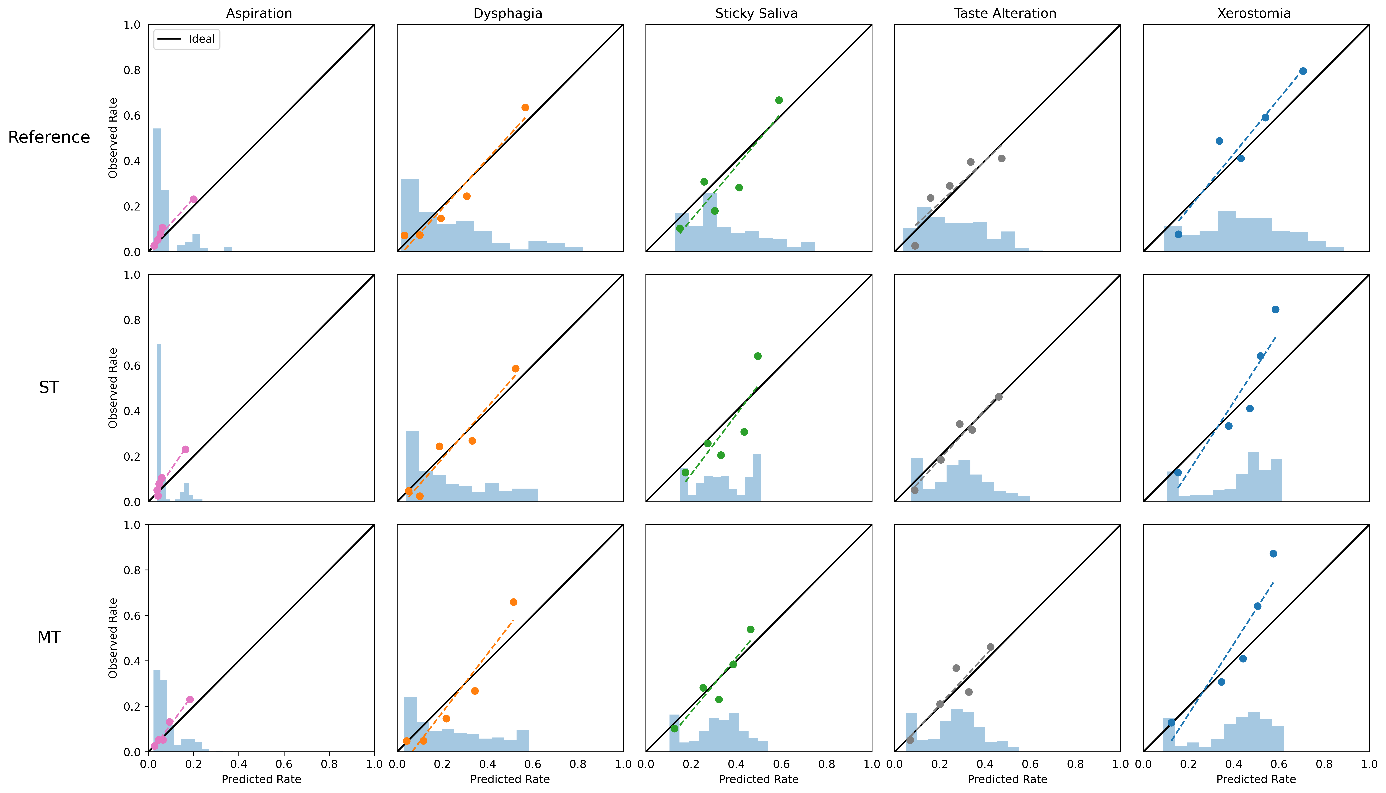


Figure F.1: Calibration plots of all models’ predictions on the independent validation cohort. Abbreviations: ST = single-toxicity deep learning model, MT = multi-toxicity deep learning model.


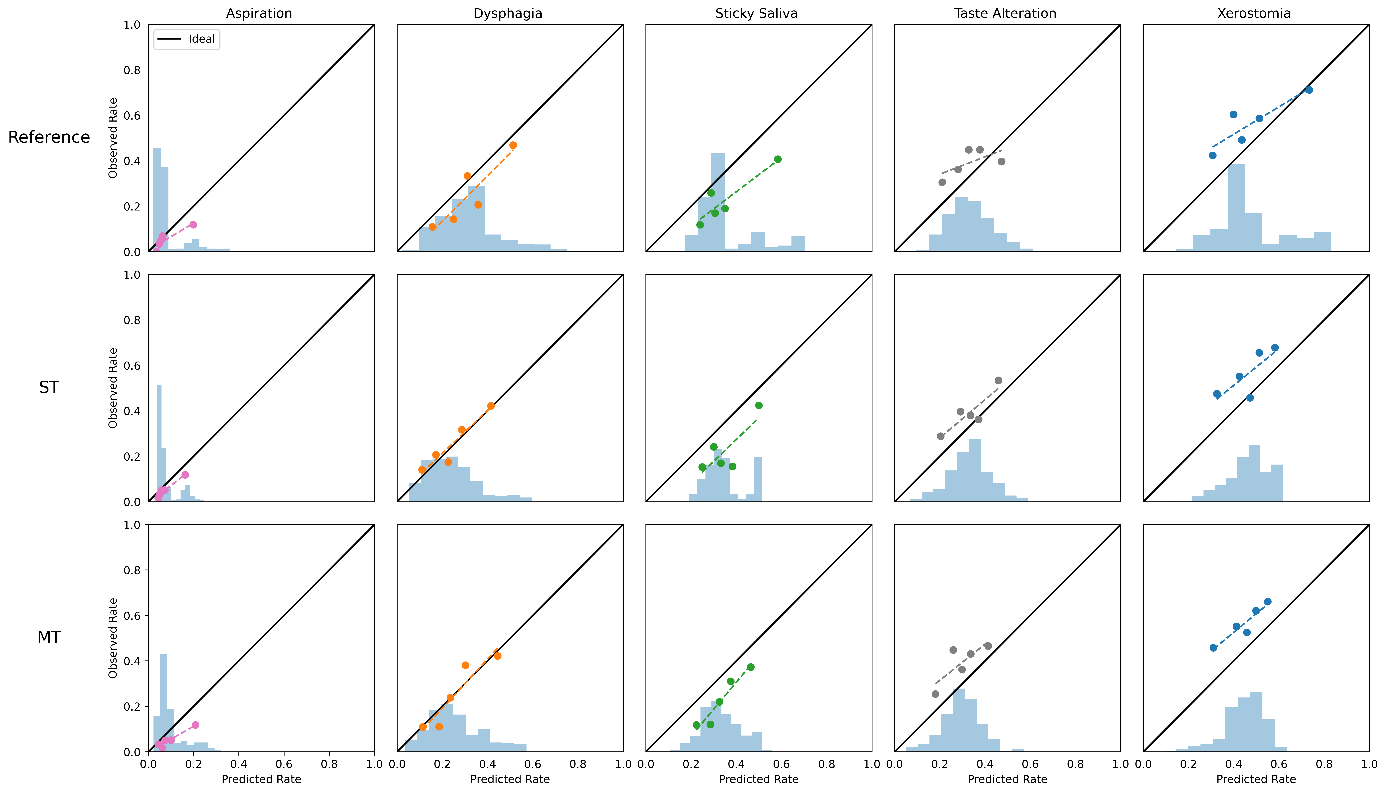


Figure F.2: Calibration plots of all models’ predictions on the external validation cohort. Abbreviations: ST = single-toxicity deep learning model, MT = multi-toxicity deep learning model.

# Appendix G: Scatter Plots of NTCP Models’ Predictions


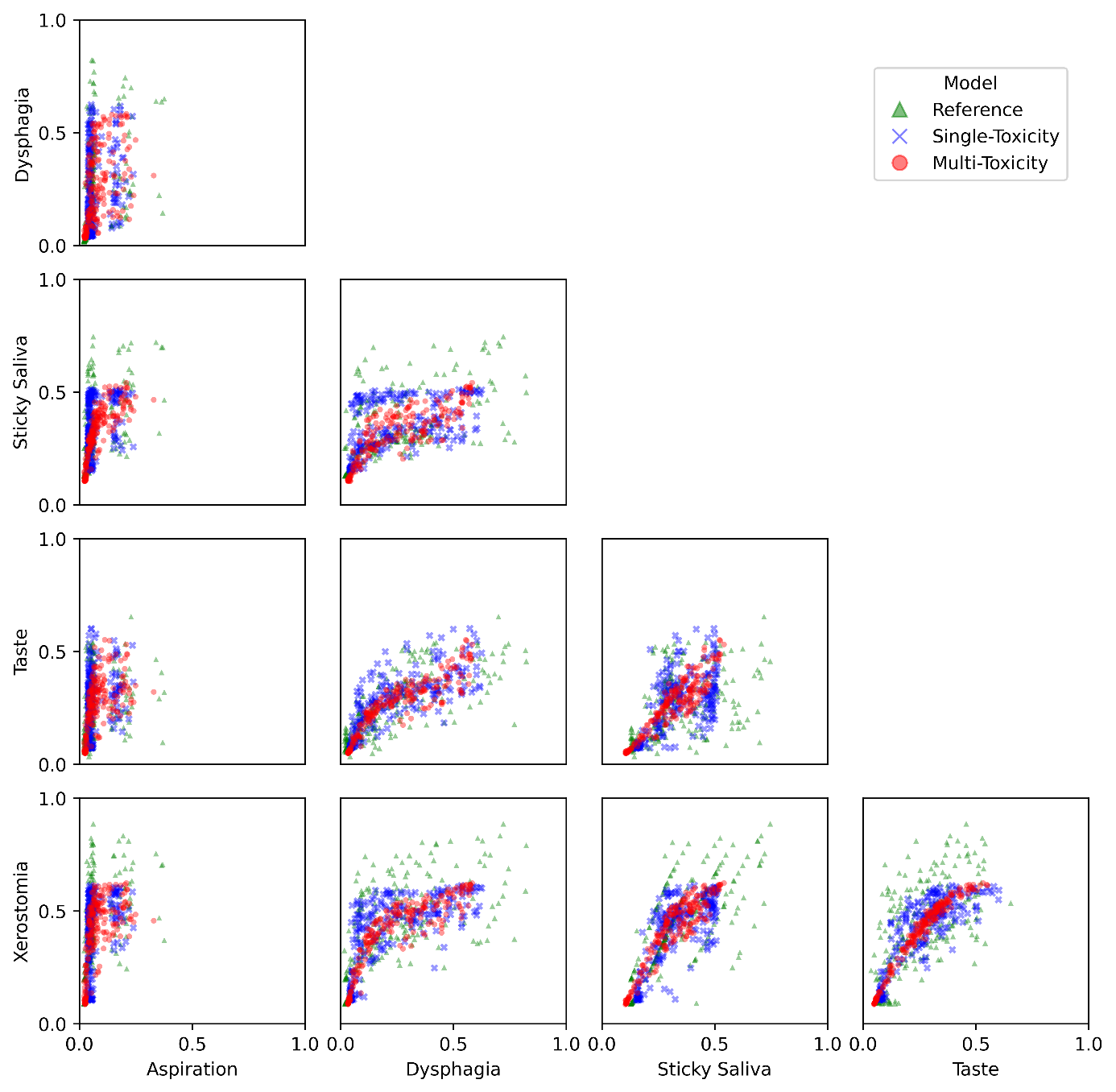


Figure G.1: Scatter plots of the predictions of all models on the independent validation cohort for each pair of the five toxicities.


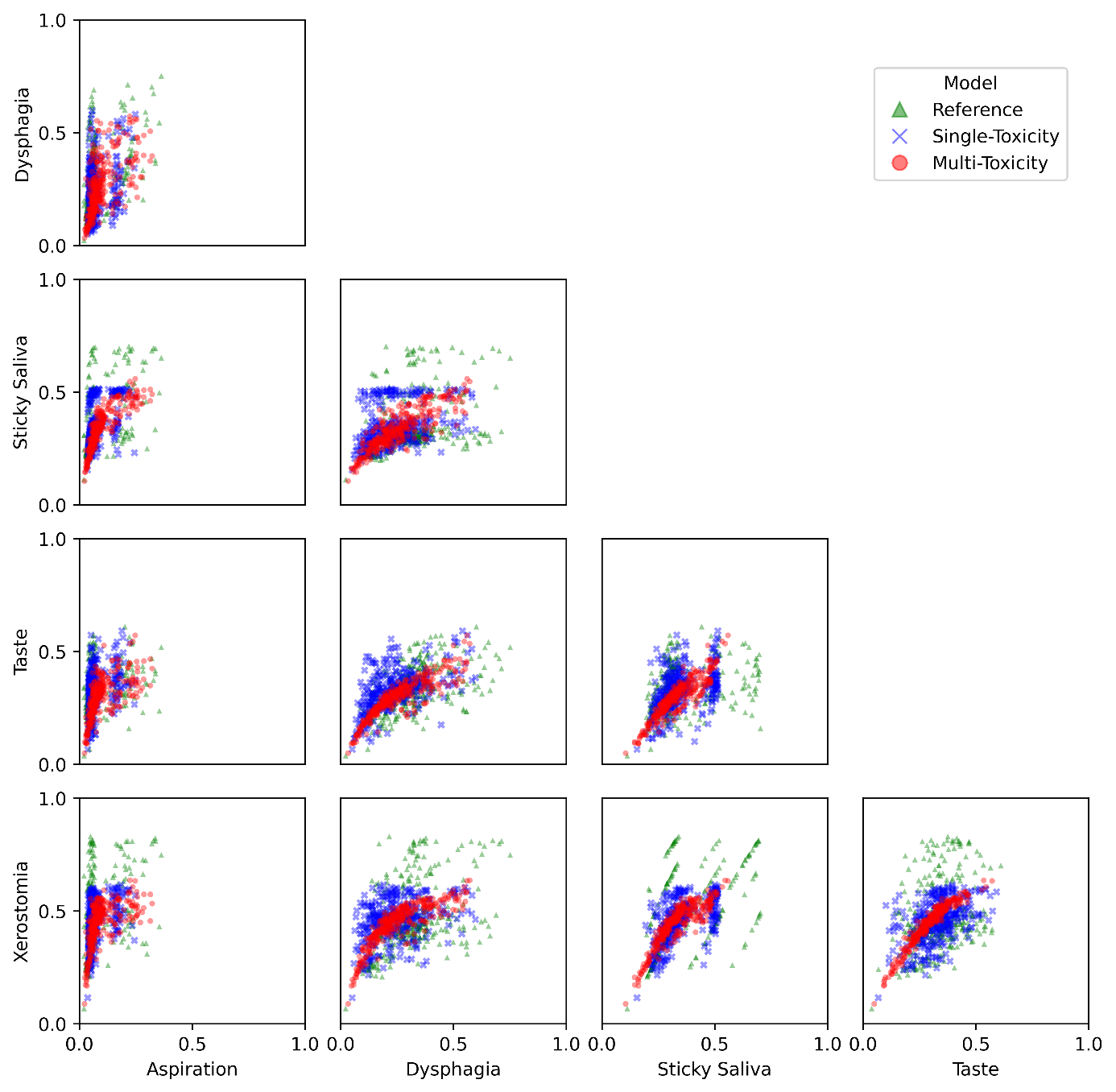


Figure G.2: Scatter plots of the predictions of all models on the external validation cohort for each pair of the five toxicities.

## References

[1] L. V Van Dijk *et al.*, “Improving automatic delineation for head and neck organs at risk by Deep Learning Contouring,” *Radiotherapy and Oncology*, vol. 142, pp. 115–123, Jan. 2020, doi: 10.1016/j.radonc.2019.09.022.

[2] M. J. Cardoso *et al.*, “MONAI: An open-source framework for deep learning in healthcare,” Nov. 2022.

[3] H. Zhang, M. Cisse, Y. N. Dauphin, and D. Lopez-Paz, “mixup: Beyond Empirical Risk Minimization,” Apr. 2018, *arXiv*. doi: 10.48550/arXiv.1710.09412.

[4] US Department of Health and Human Services National Institutes of Health National Cancer Institute, “Common Terminology Criteria for Adverse Events (CTCAE) Version 5.”

[5] D. I. Rosenthal *et al.*, “Measuring head and neck cancer symptom burden: The development and validation of the M. D. Anderson symptom inventory, head and neck module,” *Head Neck*, vol. 29, no. 10, pp. 923–931, 2007, doi: 10.1002/hed.20602.

[6] K. Bjordal *et al.*, “A 12 country field study of the EORTC QLQ-C30 (version 3.0) and the head and neck cancer specific module (EORTC QLQ-H&N35) in head and neck patients. EORTC Quality of Life Group,” *Eur. J. Cancer*, vol. 36, no. 14, pp. 1796–1807, Sep. 2000, doi: 10.1016/s0959-8049(00)00186-6.

[7] S. P. M. de Vette *et al.*, “Evaluation of a comprehensive set of normal tissue complication probability models for patients with head and neck cancer in an international cohort,” *Oral Oncol.*, vol. 163, p. 107224, Apr. 2025, doi: 10.1016/j.oraloncology.2025.107224.

[8] L. Van Den Bosch *et al.*, “Comprehensive toxicity risk profiling in radiation therapy for head and neck cancer: A new concept for individually optimised treatment,” *Radiotherapy and Oncology*, vol. 157, pp. 147–154, Apr. 2021, doi: 10.1016/j.radonc.2021.01.024.

[9] L. Van Den Bosch *et al.*, “Key challenges in normal tissue complication probability model development and validation: towards a comprehensive strategy,” *Radiotherapy and Oncology*, vol. 148, pp. 151–156, Jul. 2020, doi: 10.1016/j.radonc.2020.04.012.

[10] A. Dosovitskiy *et al.*, “An Image is Worth 16x16 Words: Transformers for Image Recognition at Scale,” Jun. 2021, [Online]. Available: http://arxiv.org/abs/2010.11929

[11] A. Paszke *et al.*, “PyTorch: An Imperative Style, High-Performance Deep Learning Library,” Dec. 2019, *arXiv*. doi: 10.48550/arXiv.1912.01703.

[12] T. Akiba, S. Sano, T. Yanase, T. Ohta, and M. Koyama, “Optuna: A Next-generation Hyperparameter Optimization Framework,” Jul. 2019, *arXiv*. doi: 10.48550/arXiv.1907.10902.
